# Arriving Safely: Decreasing Rapid Escalations in Care for Incoming Transported Pediatric Patients

**DOI:** 10.1097/pq9.0000000000000624

**Published:** 2022-12-27

**Authors:** Christie Zheng, Cynthia Gibson, Hyungjoo Jeong, Courtney Port

**Affiliations:** From the * Medical Student, University of Virginia School of Medicine, Charlottesville, Va.; † Pediatric Critical Care Physician and Chair, Department of Pediatrics, Inova Children’s Hospital, Falls Church, Va.; ‡ Department of Pediatrics, University of Virginia School of Medicine, Inova Campus, Falls Church, Va.; § Pediatric Resident Physician, Department of Pediatrics, Inova Children’s Hospital, Falls Church, Va.; ‖ Pediatric Hospital Medicine Physician, Department of Pediatrics, Inova Children’s Hospital, Falls Church, Va.

## Abstract

Accurate inpatient placement at the level of care needed for incoming transported patients can present challenges that may result in a rapid escalation in care following admission to the general inpatient unit. The Pediatric Early Warning System (PEWS) score is useful for early recognition of clinical deterioration. Therefore, we aimed to reduce rapid escalations in care for incoming transported patients via the implementation of an escalation algorithm with a goal of 80% PEWS score completion rate during transport within 6 months.

**Methods:**

We created an escalation algorithm utilizing PEWS scores and direct lines of communication between emergency medical technicians and receiving physicians. Audit and feedback increased the adoption of the process. We defined rapid escalations as transfer to a higher level of care within 6 hours of admission.

**Results:**

PEWS score completion increased from a mean of 48% to 70%. This result varied by emergency medical technician crew level of care. Eleven percent (n = 114) of PEWS scores required physician notification, 20% (n = 23) of which resulted in interventions en route. There were no differences in rapid escalation rates over time, but it remained low at <2% of all incoming transported patients. Some crew members report improved communication with hospital providers and feel more empowered to speak up when a patient’s assessment is not as expected following algorithm implementation.

**Conclusions:**

This project improved PEWS score completion and maintained a low rate of rapid escalations of care among incoming transfers.

## INTRODUCTION

Accurate inpatient placement for the level of care needed is crucial for good patient outcomes. Escalation to the intensive care units (ICUs) from general adult wards is shown to have higher mortality than direct admissions from the emergency departments (EDs).^1–4^ Similarly, in the pediatric population, indirect admissions to the pediatric ICUs (PICU) have higher mortality, a longer length of stay, and more significant interventions performed.^5,6^

Determining the correct disposition before hospital admission can be challenging, especially in patients transported from outlying facilities. Children often present to outlying facilities that lack pediatric expertise, which may result in less appropriate treatment, inaccurate assessments, and/or poor communication with accepting facilities.^7–9^ Additionally, there can be changes in clinical status due to illnesses with rapid progression or prolonged transfer times because of distance traveled or availability of transport crews. Correct placement requires flexibility, continuous monitoring, and direct communication between sending providers, emergency medical technician (EMT) crews, and receiving providers to achieve the best final disposition. Some children’s hospitals work around this issue by routing all incoming transported admissions through their pediatric ED. Doing so, however, may not be the best utilization of ED beds and staff.

The Pediatric Early Warning Score (PEWS) is useful for early recognition of deterioration in clinical status. It consists of three categories, behavior, cardiovascular, and respiratory, allowing easy calculation. Initially used in the inpatient setting, PEWS has been increasingly studied outside the wards to assist patient disposition. Several studies utilizing PEWS within the ED found that higher PEWS is associated with the need for a higher level of care.^10–13^ Using PEWS in the ED has an excellent interrater reliability between ED RNs,^10^ and moderate agreement between physicians and trainees^11^ may also increase provider communication.^10–13^ Transport PEWS (TPEWS) was also developed for monitoring illness severity during interfacility transport. Studies have shown that higher TPEWS were more likely to require PICU admission and suggested that the score can provide a common language among different facilities.^7,14^

This study aimed to reduce rapid escalations in the care of incoming transported pediatric patients via implementing a PEWS system and escalation algorithm with a goal of 80% of patients having PEWS completed during transport within 6 months.

## METHODS

### Data Collection

This single-center study took place in a Mid-Atlantic suburban children’s hospital with 226 pediatric beds, an average of 7,000 general pediatric and 900 PICU admissions per year, and approximately 1,600 incoming transported admissions per year. Project team members included a resident physician, the pediatric quality chair, a hospitalist QI expert, clinical logistics coordinators, and the transport service director. In addition, our institution’s contracted transport provider has EMT teams and may have specific pediatric transport teams, including local pediatric advanced life support (PALS) crews and advanced cardiovascular life support (ACLS) crews.

The transport supervisor collected data, including the initial, transporting, and arrival PEWS scores, calls to receiving physicians, interventions made after discussing with receiving physicians, and changes to arrival location determined en route. Data were sent to the quality improvement (QI) team every one to three months. In addition, we identified rapid escalations after admission from our hospital’s safety reporting system, through which reporting by nursing leaders of a patient’s transfer to a higher level of care is mandatory. We define rapid escalation as patients requiring transfer from the general pediatric ward to the intermediate care unit (IMC) or ICU within 6 hours of admission. We chose a 6-hour threshold to reflect the impact of the initial placement more closely. Variables, including time and reasons for escalation, were collected from the electronic medical records and linked to matching transport data.

### Subjects

From September 2019 to December 2021, patients <18 years old admitted to the general pediatric units via prehospital patient transport were included in the study. We excluded patients requiring intubation or sedation, admitted to the neonatal ICU, admitted directly from the hospital’s ED or operative room, ED to ED transfers, and patients transported by basic life support (BLS) crews.

### Interventions

**Fig. 1.**
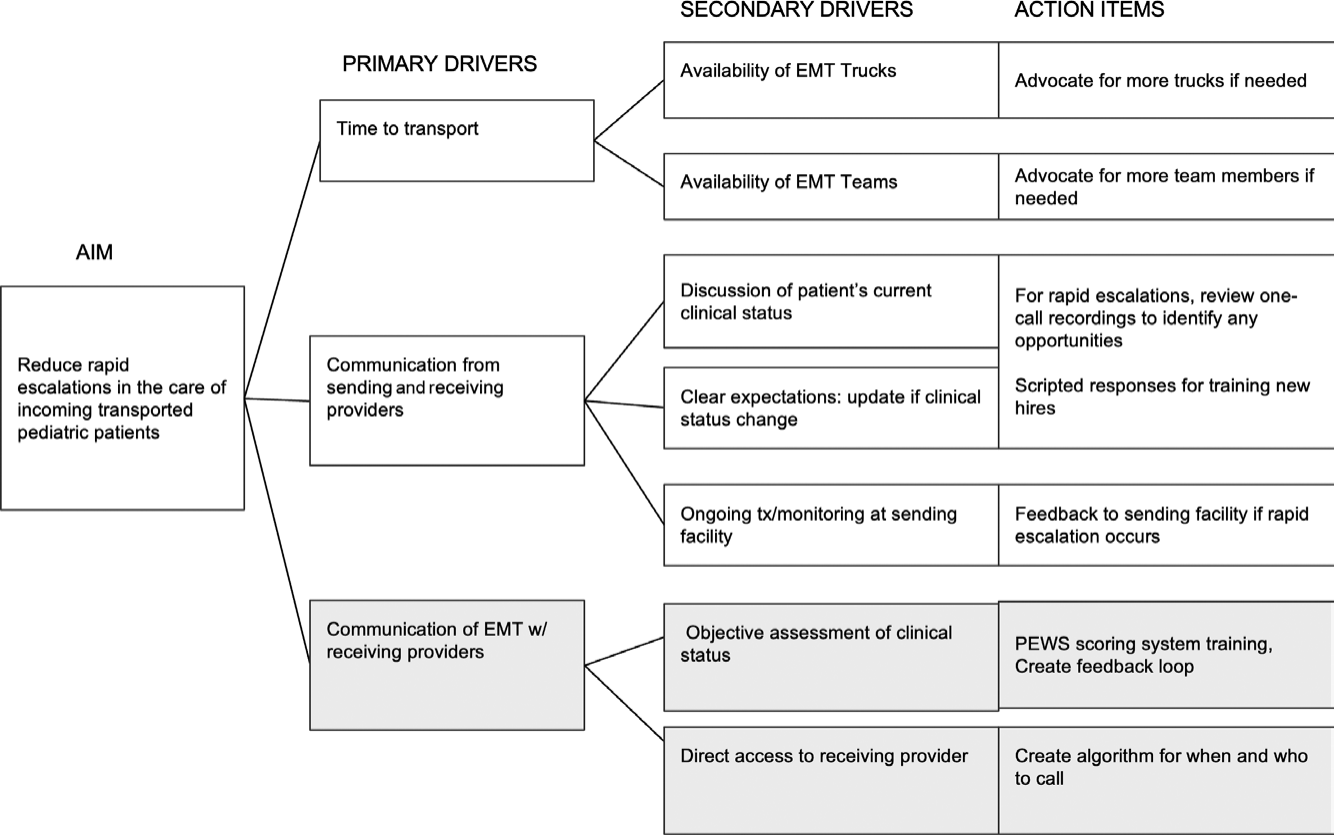
Project driver diagram. Team members focused on the highlighted drivers for this project.

**Fig. 2.**
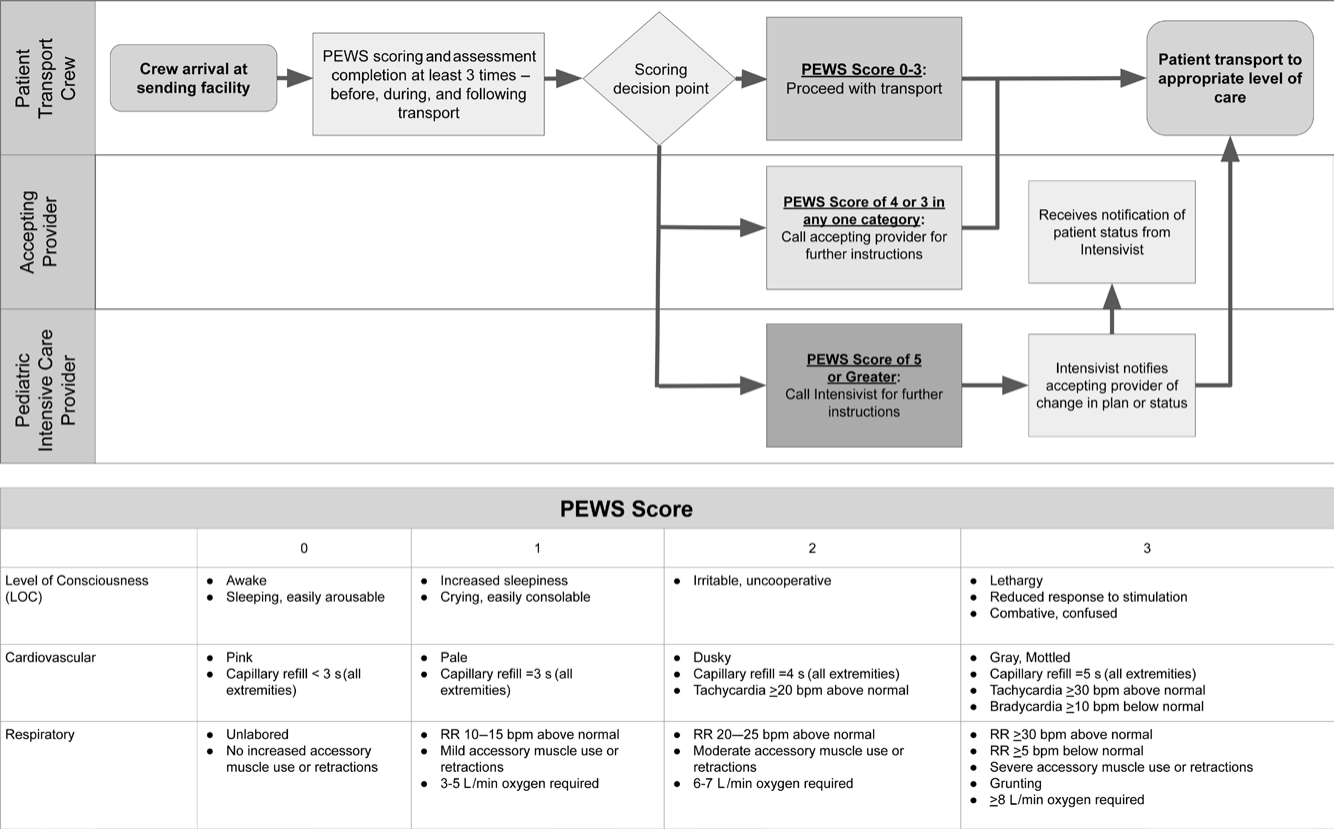
Patient transport escalation algorithm with the PEWS.

QI team members created a key driver diagram to identify key drivers and action items to minimize rapid escalation (Fig. 1). We created an escalation algorithm using the PEWS scoring system (Fig. 2). This process requires PEWS scoring at three time points—upon arrival at the sending facility, during transport, and arrival at the receiving facility. We used PEWS instead of TPEWS to ensure consistency between the transport crews and our inpatient units. A system for direct communication between EMT crews and accepting physicians was established and formalized, including providing crew members with the receiving pediatric hospitalist physicians’ direct phone number that was carried on their person throughout their shift, thus enabling immediate contact. Thresholds for contacting the receiving provider include a total PEWS score of four or three in any category. The threshold for contacting the intensivist is a total PEWS score of five or greater. The QI team member and intensivist (C.G.) taught EMT crews the PEWS scoring system and the algorithm workflow via a lecture. We provided presentation slides from this lecture to the transport providers for review and future onboarding. The QI team used audit and feedback to increase the adoption of the process. We utilized the Model for Improvement and PDSA cycles.

### Evolution of Interventions over Time

The initial implementation of the escalation algorithm and PEWS scoring system was in September 2019 but was quickly put on hold, and data collection was paused due to the COVID-19 pandemic. The QI team restarted this project in 2021 and added additional team members to assist with data collection, analysis, and intervention development. The team focused on optimizing the communication of PEWS score audit results and feedback to the EMT crews. Initially, we sent results to the transport supervisor without direct means to communicate with the crew members. We then requested that the results be emailed to crew members, posted in workrooms, and discussed at staff meetings. Finally, we created a standardized process to send the results each month. Project results were also presented during hospitalist staff meetings.

### Measures

The primary outcome measure was the rate of rapid escalations over time, calculated as the number of rapid escalations divided by total transported general pediatric admissions. We also calculated the number of general pediatric transports between each rapid escalation. We compared the median transport time, calculated as the destination arrival time minus the time at which the EMT crew was ready to pick up the patient in minutes, for patients with rapid escalations and all other transported patients. The main process measure was the percentage of incoming transported patients with completed PEWS scores by EMT crews. We also measured the percent agreement between the arrival PEWS score by EMT crews and arrival PEWS score by bedside RNs for patients who required rapid escalation in care, as these patients were felt to be least likely to have a good interrater reliability. Raw score difference was calculated, as well as categorical differences consistent with the escalation algorithm (mild = 0–3, moderate = 4, and severe *≥* 5). Balancing measures include the satisfaction of EMT crew members and the percentage of patients with completed PEWS scores who require a phone call to the receiving physicians. We measured EMT satisfaction with a survey using a five-point Likert scale.

### Analysis

We analyzed data on PEWS completion for transported admissions and rapid escalations from January to December 2021. We created statistical process control charts in Excel using QI Macros. Centerline adjustments were made when established control chart rules were met. Upper and lower control limits were defined as >3 standard deviations above or below the mean. We used the Montgomery rules to define special-cause variation.^15^ A g-control chart analyzes the number of transported admissions between rapid escalations. A p-control chart displays PEWS completion with each data point containing data from 50 consecutive patient transfers. We used Chi-Square testing to analyze the difference in PEWS score completion among different crew types. We used independent T-testing to compare transport durations between patients with and without rapid escalations.

### Ethical Considerations

This project was a QI activity and was exempt from our local institutional review board.

## RESULTS

**Fig. 3.**
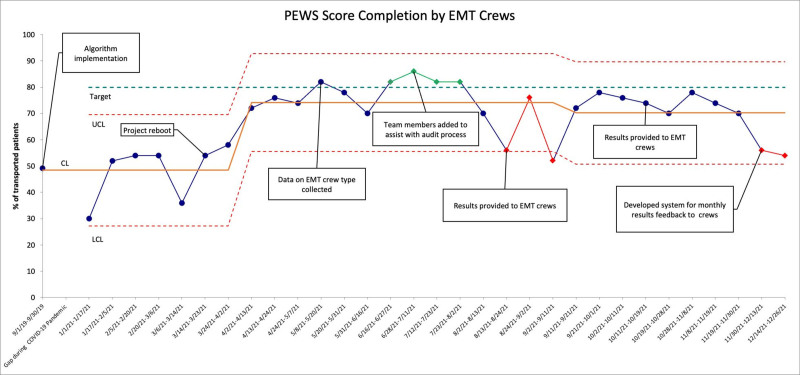
*P* control chart showing the percentage of incoming transported patients with PEWS score completion by EMT crews over time. Green data points are unstable points showing favorable changes, and red data points show unfavorable changes. The gap in data collection during the COVID-19 pandemic occurred in the year of 2020. CL, centerline; LCL, lower control limit; UCL, upper control limit.

Within the study period, there were a total of 1,544 transfers. Of which, 1,039 (67%) had PEWS completed by EMT. PEWS completion was initially 54% (31/57 transports) in the first month following algorithm implementation. After a pause in the project due to the COVID-19 pandemic, the mean decreased to 49% but following targeted interventions increased to 74% (Fig. 3). PEWS completion rates varied significantly by the crew type, with 79% (n = 599) for our local PALS transport team and 58% (n = 187) for ACLS (*P* < 0.00001). Eleven percent (11%, n = 114) of PEW assessments required physician notifications. Of these transfers, 20% (n = 23) changed management en route after discussion with the receiving physicians. There was one de-escalation determined en route. Before the implementation of the escalation algorithm, EMT crews were not routinely provided phone numbers for the accepting physicians. We found categorical agreement in PEWS score (mild = 0–3, moderate = 4, and severe *≥* 5) between EMT crews and bedside RNs on arrival in 75% (n = 6) of the patients that required rapid escalation in care. In all instances, the total scores differed by no more than one point.

**Fig. 4.**
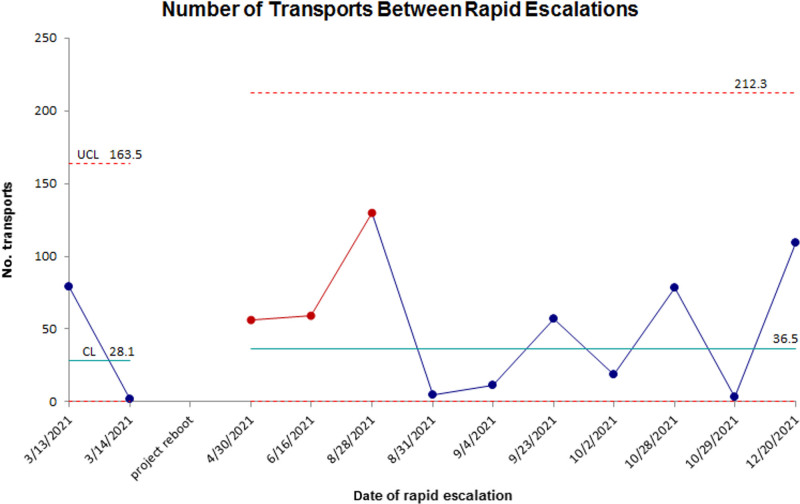
G control chart showing the number of incoming transported admissions between each rapid escalation over time during the study period. Red data points showing favorable changes correspond to when PEWS score completion was the highest. The centerline was adjusted following the process change. CL, centerline; LCL, lower control limit; UCL, upper control limit.

Despite the fluctuations in PEWS score completion, the percentage of transported patients requiring rapid escalations after admission to the ICU or IMC remained below 2% of incoming transfers per quarter during the study period ranging from 1.1% (n = 2) to 1.7% (n = 4) (*P* = 0.9). The mean number of transports between rapid escalations was 28.1 before the project reboot in March 2021 and 36.5 afterward (Fig. 4). There were 12 (0.8%) rapid escalations of transported patients in 2021, and 33% (n = 4) did not have PEWS scores completed during transport. Of the eight (67%) patients with PEWS scores completed, 63% (n = 5) required EMTs to contact the receiving physicians per the algorithm. Upgrades were attempted during transport for three of these patients but were too close to adjust arrival location (n = 2) or were denied by the accepting physician (n = 1). Interestingly, 83% (n = 10) of rapid escalations occurred on night shifts (7 pm–7 am), and 80% (n = 8) of these escalations occurred after midnight. Fifty-eight percent (58%, n = 7) of rapid escalations were due to increased respiratory support needs, and 25% (n = 3) were due to neurologic issues, including status epilepticus and altered mental status. Patients with rapid escalations had a 2.33 hours longer median transport time compared to other transports (5.98 versus 3.67 hours), but this difference was not significant (*P* = 0.18).

Unfortunately, contract renewal with the transport service was not pursued at the end of the study period, and the response rate to the EMT satisfaction survey was low. However, the two responses agreed that the PEWS system and the escalation algorithm improved communication between EMT crews and the receiving physicians. There have also been verbal reports from EMT crews that the algorithm empowered them to speak up when a patient’s assessment was not as expected.

## DISCUSSION

This project aimed to create a system to safely admit and accurately place incoming transported patients and reduce the rate of rapid escalations in care after admission. This project achieved improvement by increasing the PEWS score completion rate from a mean of 49% to 70% but has not yet achieved its goal of an 80% completion rate. Although unchanged, the rate of rapid escalations of transported patients remained below 2% throughout the project. Therefore, the lack of reduction in rapid escalations may be due to the already low rate.

Our study adds to the literature on using PEWS scores outside the ward environment, specifically on using PEWS scores in transported patients. Although there are previous studies using TPEWS, data on using PEWS during transport are currently scarce. To the best of our knowledge, our study has the largest sample size for using PEWS for transported patients. In addition, it is the first to describe an escalation algorithm with clear expectations and objective thresholds for contacting receiving physicians. Doing so created a direct line of communication between transport crews and the receiving physicians. Although the response rate to the EMT satisfaction survey was low, there were reports from crew members expressing improved communication after implementing the algorithm.

Similarly, Petrillo-Albarano et al.^7^ suggested that TPEWS during transport can serve as a common language among facilities and team members to better convey severity and increase communication clarity. Improved communication can have positive impacts outside the targeted goals, such as reducing healthcare costs and increasing mutual respect in a multidisciplinary team and staff satisfaction.^16^ Dúason et al.^17^ interviewed a group of EMT crews, nurses, and physicians at EDs and reported that such structured communication and effective feedback could be helpful for the professional development of team members.

Our study’s rate of rapid escalation to IMC and ICU units was low, and the sample size was too small for statistical analysis of the corresponding PEWS during transport. Patel et al.^18^ showed a trend that higher PEWS during transport were associated with PICU transfers, although not statistically significant. Other studies using TPEWS have also reported similar findings.^7,14^ Specifically, Petrillo-Albarano et al.^7^ suggested that patients with TPEWS of six or higher should be assessed for PICU admission, which corresponds to our threshold of five or higher for contacting the intensivists.

The majority of the rapid escalations in our study occurred during night shifts, mostly after midnight. Other authors have reported higher incidences of nighttime escalation of care, with worsening respiratory status as the leading cause.^19,20^ We hypothesized that the predominance of rapid escalations at night might be due to reduced staffing, including fewer attending physicians compared to the daytime. A previous qualitative study also cited differences in senior physician staffing and workload variability as factors impacting the timeliness of escalation of care.^21^ Additionally, two rapid escalations occurred despite EMT request for an upgrade during transport because the patient was too close to adjust the arrival location. While we are unaware of ICU bed availability during these two upgrade requests, future improvement efforts may be needed to adjust a patient’s arrival location rapidly.

Our study has several limitations. Because this is a single-institution study, results may not be generalizable. Although mandatory, the process of identifying rapid escalations relies on staff remembering to report them; therefore, some patients with escalations in care may be missed. We could also not alter any EMT crew forms to include PEWS, as the forms were nationally standardized across the company. All PEWS during transport, therefore, had to be collected by hand using supplemental forms. Previous studies show that automated PEWS improves the accuracy of scoring.^22,23^ In addition, the relatively uncommon event of a PEWS score requiring physician notification would likely have benefited from other system support to integrate the process into the daily workflow and ensure its use. We were unable to make such changes within the transport company. Last, our institution did not renew the contract with the transport service, which likely impacted the PEWS score completion rates at the end of the project. We were also not able to implement additional interventions such as partnering with our call center to remind crew members to perform PEWS scores and improving communication between the sending and the receiving physicians, including a standardized patient handoff that sets clear expectations for ongoing treatments, monitoring, and notifications of clinical change.

## CONCLUSIONS

We improved the percentage of transported patients with PEWS score completion by implementing an escalation algorithm and direct communication between EMT crews and receiving physicians. The rate of rapid escalations remained less than 2% throughout the study period. Crew members reported improved communication between transport crews and hospital providers. More research is needed to determine if the use of PEWS for transported patients can reduce rapid escalations in care.

## ACKNOWLEDGMENTS

Presented in *Pediatric Hospital Medicine* in July 2022, and in Pediatric Academic Society in April 2022.

## DISCLOSURE

The authors have no financial interest to declare in relation to the content of this article.
